# Role of endoscopic esophageal dilation in managing eosinophilic esophagitis: A systematic review and meta-analysis

**DOI:** 10.1097/MD.0000000000005877

**Published:** 2017-04-07

**Authors:** Harsha Moole, Kavitha Jacob, Abhiram Duvvuri, Vishnu Moole, Sowmya Dharmapuri, Raghuveer Boddireddy, Achuta Uppu, Srinivas R. Puli

**Affiliations:** a Department of Medicine, University of Illinois College of Medicine at Peoria, Peoria, IL; b Department of Medicine, Apogee physicians, Genesis Medical Center, Davenport, IA; c Division of Gastroenterology and Hepatology, Kansas City Veteran Affairs Medical Center, Kansas City, MO; d Department of Medicine, NTR University of Health Sciences, Vijayawada, Andhra Pradesh, India; e Department of Gastroenterology and Hepatology, Emory University School of Medicine, Atlanta, GA; f Division of Gastroenterology and Hepatology, University of Illinois College of Medicine at Peoria, Peoria, IL.

**Keywords:** clinical improvement, complications, endoscopic esophageal dilation, eosinophilic dilation, systematic review and meta-analysis

## Abstract

**Background:**

Eosinophilic esophagitis (EoE) is a chronic, immune-mediated disorder of the esophagus characterized by mucosal eosinophilic infiltration. Topical glucocorticoids are considered standard line of treatment, whereas endoscopic dilations are performed for patients presenting with treatment-resistant disease or manifestations of dysphagia and/or food impactions. Efficacy and safety of esophageal dilation in these patients are currently unclear.

**Aims:**

Primary outcomes were to evaluate the efficacy, adverse events, and mortality rates of endoscopic esophageal dilation in patients with EoE.

**Methods: Study Selection Criteria:**

Studies that reported the use of esophageal dilation in EoE patients were included in this meta-analysis.

**Data collection and extraction:**

Articles were searched in Medline, Pubmed, and Ovid journals. Two authors independently searched and extracted data. The study design was written in accordance to PRISMA statement. Clinical improvement was defined as patient-reported symptom relief noted by the authors of individual studies. The symptoms were assessed on various nonstandardized, however, relevant questionnaires that were deemed appropriate by the senior authors of individual studies.

**Statistical Method:**

Pooled proportions were calculated using fixed- and random-effects model. *I*^2^ statistic was used to assess heterogeneity among studies.

**Results:**

Initial search identified 491 reference articles, in which 39 articles were selected and reviewed. Data were extracted from 14 studies (N = 1607) using esophageal dilation for EoE management, which met the inclusion criterion. Mean age of patients was 41years. Pooled patients included 75% males. The pooled proportion of patients that showed clinical improvement with esophageal dilations, after the median follow-up period of 12 months, was 84.95%. No procedure-related deaths were noted. The pooled proportion of patients with post procedural esophageal perforation, chest pain, hospitalization, deep mucosal tear (involving muscularis propria), small mucosal tear, and hemorrhage were 0.61%, 0.06%, 0.74%, 4.04%, 22.32%, and 0.38% respectively. *I*^2^ (inconsistency) was 0% (95% confidence interval [CI] = 0–49.8) and Egger: bias was 0.06 (95% CI = −0.30 to 0.42).

**Conclusions:**

In patients with conformed diagnosis of EoE, endoscopic esophageal dilation seems to be an effective and safe treatment option. Majority patients with chest pain and deep mucosal tears did not require hospitalization and symptoms were self-limiting

## 1 Introduction

Eosinophilic esophagitis (EoE) is a chronic, immune-mediated disorder of the esophagus characterized by mucosal eosinophilic infiltration and associated clinical symptomatology in the absence of pathological gastroesophageal reflux disease (GERD).^[1]^

Although initially considered a manifestation of GERD, it has gained recognition over the years as a distinct entity defined as an allergen-mediated process in which eosinophils are recruited to the esophagus resulting in a count of ≥15 eosinophils per high-power field. Current lines of evidence support the role that EoE is induced by food allergens and primarily mediated by Th2 cell activity.^[1]^ Symptoms typically manifest in adolescents and young adults as dysphagia, heart burn, and food impaction.^[1–3]^

Early endoscopic findings include linear furrows, strictures, and esophageal rings. Persistent inflammation can lead to remodeling and fibrosis. This can result in reduced wall compliance, esophageal and esophageal narrowing, which can manifest as dysphagia and food impaction. EoE has not been associated as a premalignant condition.^[1–5]^

Long-term maintenance treatment in EoE has remained a debated question over the recent years. Given the chronicity of the disease and the risk of recurrence of symptoms, long-term maintenance treatment has been considered necessary in patients with evidence of chronic remodeling or severe symptoms. Current treatment modalities include elimination diet therapy, swallowed glucocorticoids, and endoscopic dilation. Thus, topical glucocorticoids are considered standard line of treatment, whereas endoscopic dilations are performed for patients presenting with treatment-resistant disease or manifestations of dysphagia and/or food impactions. Dilation remains an accepted treatment modality in the case of dysphagia secondary to esophageal strictures or esophageal narrowing.^[1]^ Previous studies have advised caution with dilation given concerns for possible perforation and post-endoscopic dilation chest pain. Recent studies, however, have shown dilation to be a safer procedure than previously described.^[4,6,7]^

Previous meta-analysis in 2013 by Moawad et al,^[4]^ analyzed the utility of endoscopic esophageal dilation in EoE patients. However, only 9 studies were included in that analysis. There were multiple well-performed studies that were published after their meta-analysis. This new information may have a role in changing the current perception of endoscopic dilation, and has to be integrated with already available evidence. Given this, we performed a comprehensive meta-analysis of all the available studies to further assess the clinical response and possible complications of esophageal dilation in EoE patients.

## 2 Methods

### 2.1 Aims

The aims of this meta-analysis are to pool the evidence for endoscopic esophageal dilation in EoE patient. Primary outcomes were to evaluate efficacy (clinical improvement in EoE patients), adverse events (post procedural esophageal perforation, chest pain, hospitalization, deep mucosal tear involving muscularis propria, small mucosal tear, and hemorrhage), and mortality rates of endoscopic esophageal dilation in patients with EoE.

### 2.2 Study selection criteria

#### 2.2.1 Inclusion criteria

Studies that reported the use of esophageal dilation in EoE patients were included in this meta-analysis. Studies must have assessed either the efficacy or adverse events of endoscopic esophageal dilation in EoE patients.

#### 2.2.2 Exclusion criteria

Individual case reports were not included in this meta-analysis. Only full English articles were included in this analysis.

### 2.3 Data collection and extraction

The study design was written in accordance to PRISMA (Preferred Reporting Items for Systematic Reviews and Meta-Analyses) statement. Ethical approval was not necessary as the study is a systematic review and meta-analysis. Articles were systematically searched in Medline, PubMed, Ovid journals, EMABSE, Cumulative Index for Nursing & Allied Health Literature, ACP journal club, DARE, International Pharmaceutical Abstracts, old Medline, Medline nonindexed citations, OVID Healthstar, and Cochrane Central Register of Controlled Trials (CENTRAL). The search was performed for the years 1966 to July 2016. Abstracts were manually searched in the major gastroenterology journals (Gastroenterology, Gut, American Journal of Gastroenterology and Gastrointestinal Endoscopy) for the past 3 years. Study authors for the abstracts included in this analysis were contacted when the required data for the outcome measures could not be determined from the publications. Search was limited to English articles. The MeSH search headings used were “endoscopic dilation," “eosinophilic esophagitis," “efficacy," and “adverse events." The reference lists of the included studies were manually searched for any relevant publications. Two authors (KJ and HM) independently searched and extracted the data into an abstraction form. Any differences were resolved by mutual agreement. If the disagreement persisted, the final decision was made by a third author (SRP) after reviewing the relevant information. The agreement between reviewers for the collected data was quantified using the Cohen κ.^[20]^ Data were extracted from the selected studies and entered into a standardized data collection form. The following variables were recorded: name and year of study; type of study; mean patient age; male/female distribution in percentage; total number of EoE patients included; number of EoE patients that underwent esophageal dilation; total number of dilations in each study; type of esophageal dilator used; definition of clinical improvement; follow-up period in months; number of patients that had clinical improvement after dilation; percentage of patients with clinical improvement; number of esophageal perforations post procedure; number of hospitalizations required as a result of procedure related complications; number of small mucosal tears post procedure; number of deep mucosal tears post procedure; procedure related hemorrhage; procedure related chest pain; number of patients with a previously established diagnosis of EoE; number of treatment-naïve EoE patients.

### 2.4 Definitions

For the purpose of this article, EoE was defined as esophageal dysfunction-related symptoms, with eosinophil predominant inflammation (>15 eosinophils per-high power field) on an esophageal biopsy, and persistence of mucosal esophageal eosinophilia after 2 months of proton pump inhibitor trial therapy. Clinical improvement was defined as patient-reported symptom relief noted by the authors of individual studies. The symptoms were assessed on various nonstandardized, however, relevant questionnaires that were deemed appropriate by the senior authors of individual studies.

### 2.5 Quality of studies

Clinical trials designed with a control and treatment arms can be assessed for quality of the study. A number of criteria have been used to assess this quality of a study (e.g., randomization, selection bias of the arms in the study, concealment of allocation, and blinding of outcome). Jadad score was used to evaluate the quality of randomized studies. Cochrane Collaborations and the Quality of Reporting of Meta-analysis guidelines were followed to assess the quality of studies.^[21,22]^ Newcastle Ottawa Scale^[23]^ was used to assess the quality of nonrandomized studies.

### 2.6 Statistical methods

This meta-analysis was performed by calculating pooled proportions. First, the individual study proportion of pain control, ductal clearance, quality of life, among others, was transformed into a quantity using Freeman-Tukey variant of the arcsine square root transformed proportion. The pooled proportion is calculated as the back-transform of the weighted mean of the transformed proportions, using inverse arcsine variance weights for the fixed-effects model and DerSimonian-Laird weights for the random-effects model.^[24,25]^ Forest plots were drawn to show the point estimates in each study in relation to the summary pooled estimate. The width of the point estimates in the Forest plots indicates the assigned weight to that study. The heterogeneity among studies was tested using *I*^2^ statistic and Cochran *Q* test based upon inverse variance weights.^[26]^*I*^2^ of 0% to 39% was considered as nonsignificant heterogeneity, 40% to 75% as moderate heterogeneity, and 76% to 100% as considerable heterogeneity. If *P* value is >0.10, it rejects the null hypothesis that the studies are heterogeneous. The effect of publication and selection bias on the summary estimates was tested by both Harbord-Egger bias indicator^[27]^ and Begg-Mazumdar bias indicator.^[28]^ Also, funnel plots were constructed to evaluate potential publication bias.^[29,30]^ Microsoft Excel 2013 software was used to perform statistics for this meta-analysis.

## 3 Results

**Figure 1:**
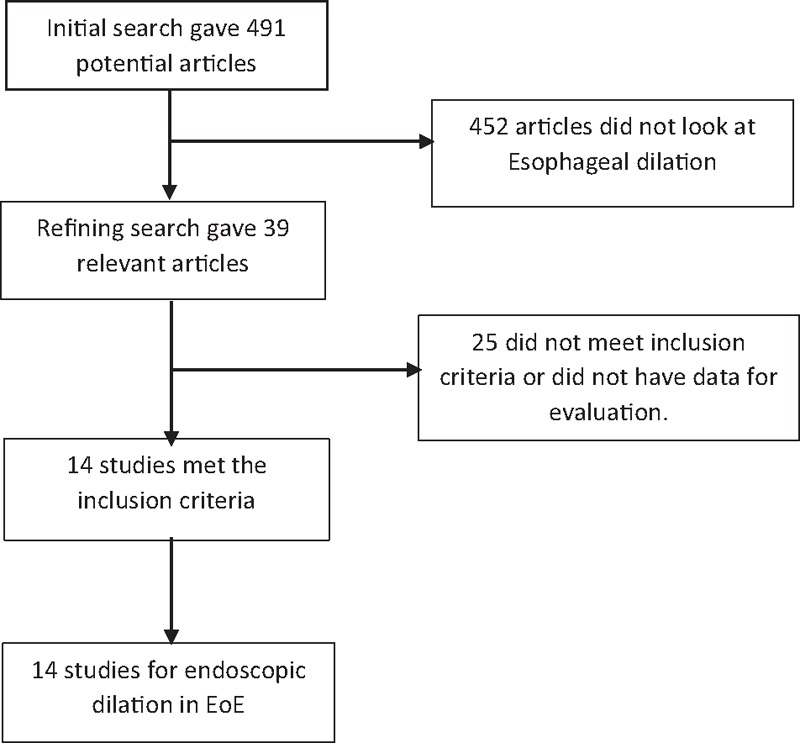
Flow diagram: Search results.

Initial search identified 491 reference articles, in which 39 articles were selected and reviewed. Data were extracted from 14 studies^[6–19]^ (N = 1607) using esophageal dilation for EoE management, which met the inclusion criterion. All the studies are published as full-text articles. Figure 1 shows the flow diagram of search results. All the pooled estimates given are estimates calculated by the fixed effect model. Random-effect model was preferred over fixed-effects model when the heterogeneity was significant. Among the 14 studies included in this analysis, 9 studies^[6,8–14,18]^ included information regarding the clinical improvement and all 14 studies included information on at least one of the adverse events associates with the procedure. Owing to the nature of data available from individual studies, we were not able to analyze and compare the different esophageal dilation techniques separately. Unfortunately, all the studies included in this analysis were retrospective studies, except one study^[14]^ that was a cross-sectional study.

The total number of patients included in this meta-analysis was 1607, with a predominantly male population (75%). Mean age of the patients was 41 years. Among the 1607 patients with a confirmed diagnosis of EoE, 809 patients underwent esophageal dilation at least 1 time. Total number of dilation procedures was 1543. Median number of dilations per patient was 2. One of the 3 techniques (savary, Maloney, and through the scope balloon) was used to perform dilation. Table 1 shows the baseline characteristics of the studies.^[6–19]^ The *P* for *χ*^2^ heterogeneity for all the pooled accuracy estimates was >0.10. The agreement between reviewers for the collected data gave a Cohen κ value of 1.0.

**Table 1:**
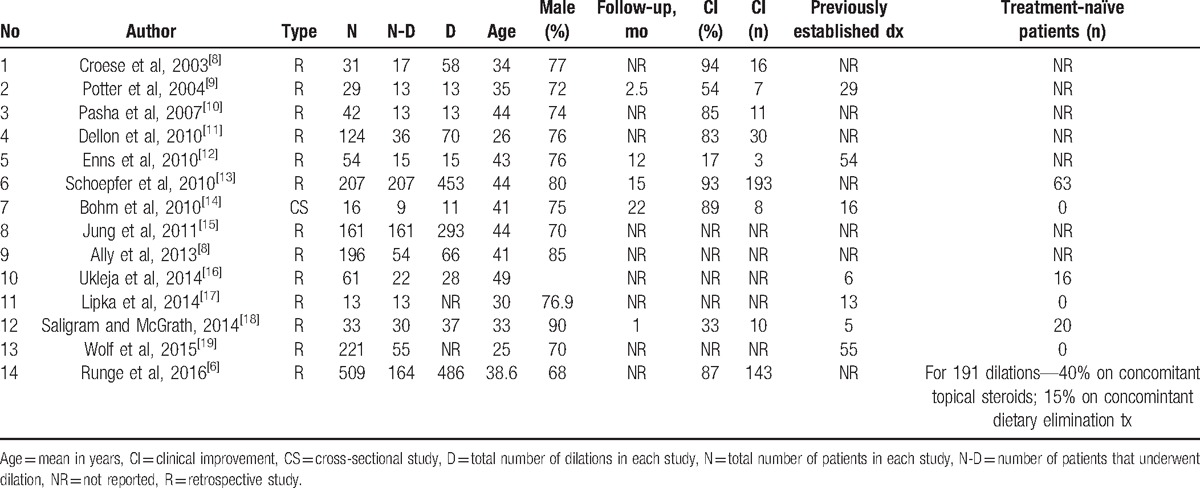
Basic characteristics of the included studies.

### 3.1 Efficacy of endoscopic esophageal dilation in EoE patients

**Figure 2:**
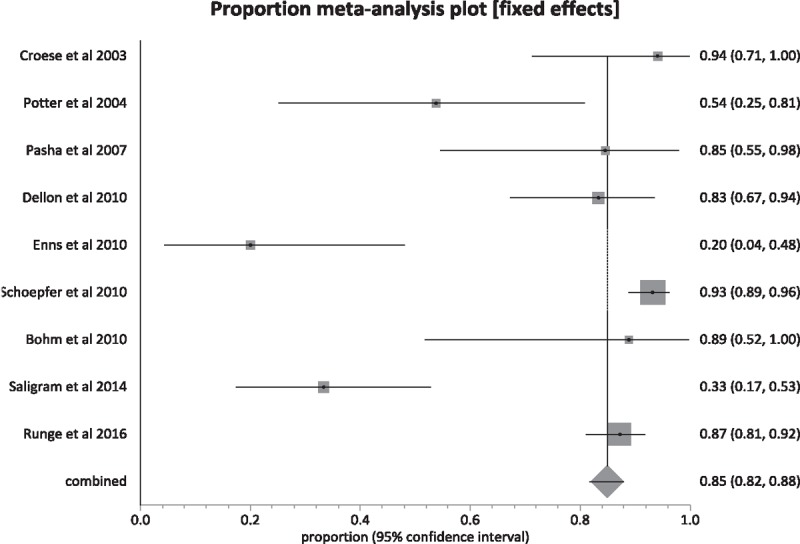
Forest plot. Individual study proportions and the pooled estimate for eosinophilic esophagitis patients with clinical improvement after endoscopic dilation.

**Figure 3:**
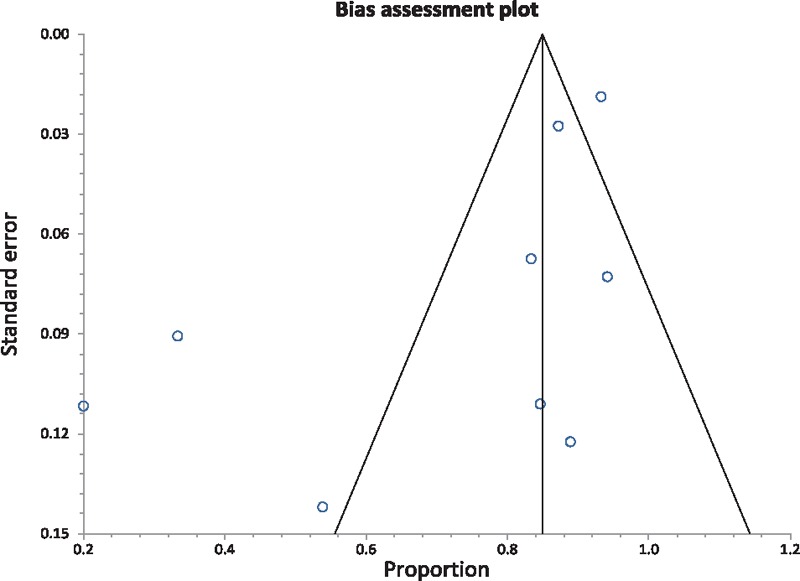
Funnel plot assessing for publication bias (for clinical improvement in eosinophilic esophagitis patients).

Data regarding clinical improvement was available in 9 of 14 studies. Definitions of clinical improvement used by individual studies are mentioned in Table 2.^[6–19]^ The pooled proportion of patients that showed clinical improvement with esophageal dilations, after the median follow-up period of 12 months, was 84.95% (95% confidence intervals [CI] = 81.72–87.93). Bias indicators for this variable: Egger, bias was −3.3 (95% CI = −6.52 to −0.02) *P* = .048. Heterogeneity for this variable was assessed using *I*^2^ (inconsistency) = 91% (95% CI = 85.6%–93.7%). Figure 2 is a forest plot representing the pooled and individual proportion of clinical improvement in EoE patients after endoscopic esophageal dilation. Figure 3 is a funnel plot assessing the publication bias for same variable.

**Table 2:**
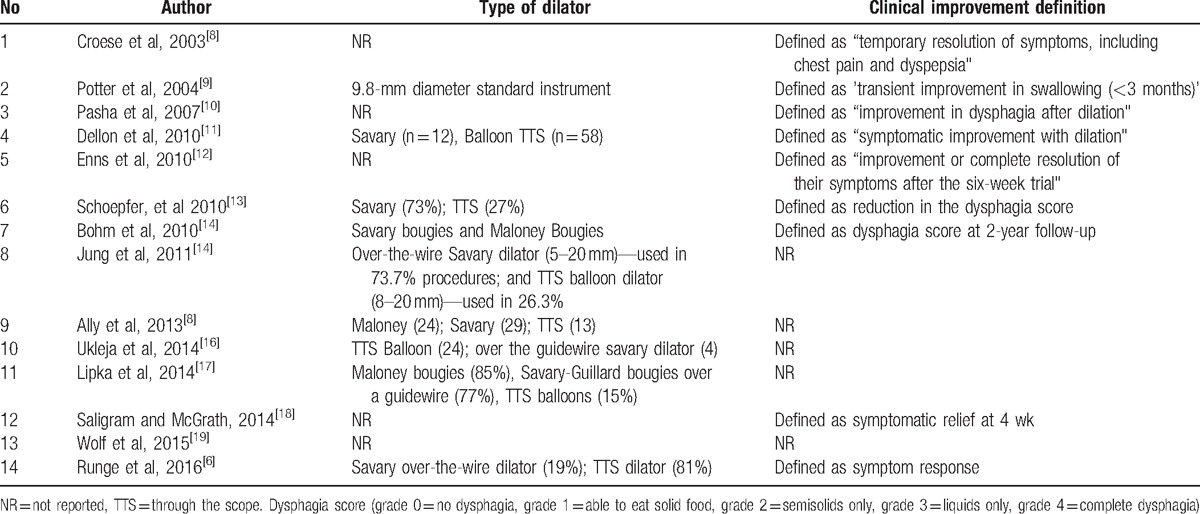
Type of esophageal dilation and clinical improvement definition in each study.

### 3.2 Safety of endoscopic esophageal dilation in EoE patients

Mortality data were available from all 14 studies included in this analysis. There were no procedure-related deaths, among the patient included in this analysis who underwent endoscopic esophageal dilation.

**Figure 4:**
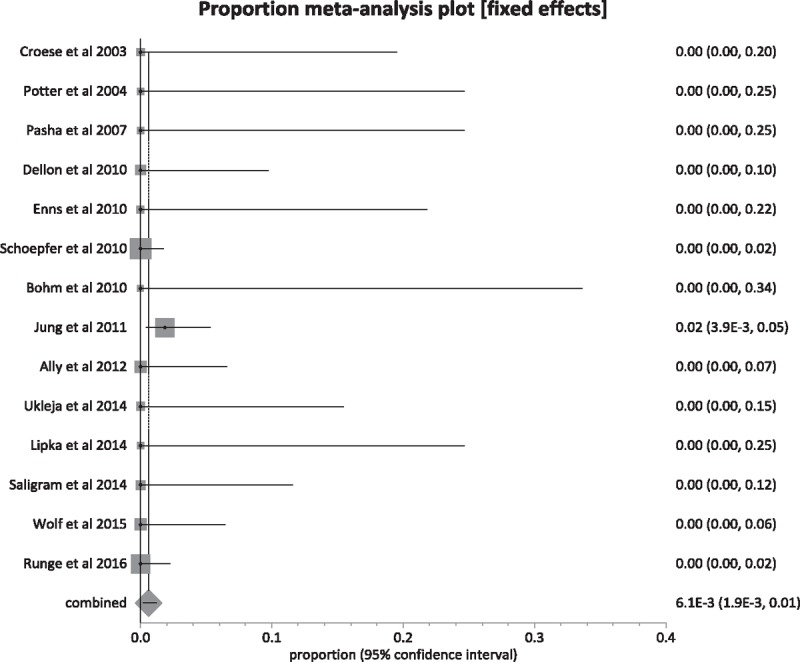
Forest plot. Individual study proportions and the pooled estimate for postprocedural esophageal perforation.

**Figure 5:**
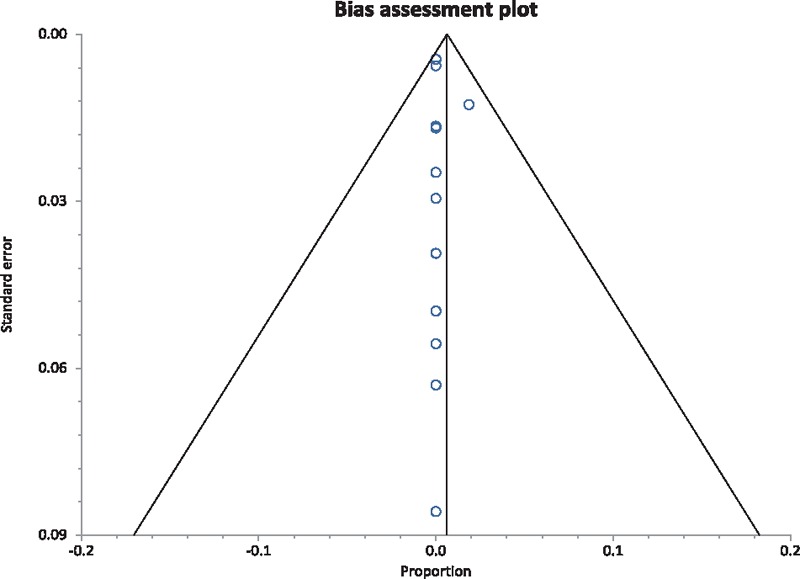
Funnel plot assessing for publication bias (esophageal perforation).

Procedure-related adverse events that were studied in this analysis were post-procedural esophageal perforation, chest pain, hospitalization, deep mucosal tear (involving muscularis propria), small mucosal tear, and hemorrhage. Table 3 includes complications from individual studies.^[6–19]^Table 4 included pooled percentage of individual complications, along with bias and heterogeneity details.^[6–19]^ The pooled proportion of patients with post-procedural esophageal perforation, chest pain, hospitalization, deep mucosal tear, small mucosal tear, and hemorrhage were 0.81%, 7.06%, 0.74%, 4.04%, 22.32%, and 0.38% respectively. Figure 4 is a forest plot representing the pooled and individual proportion of postprocedure esophageal perforation in EoE. Figure 5 is a funnel plot assessing the publication bias for same variable.

**Table 3:**
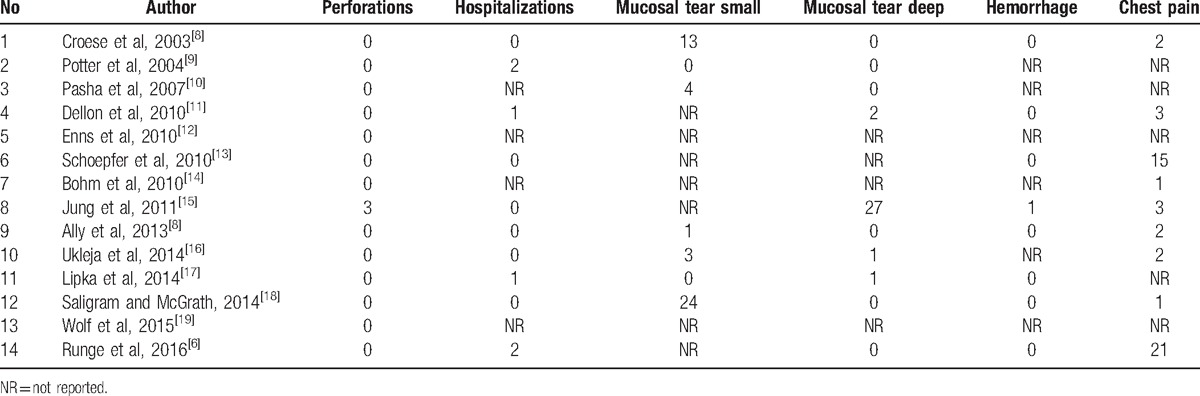
Number of complications in individual study.

**Table 4:**
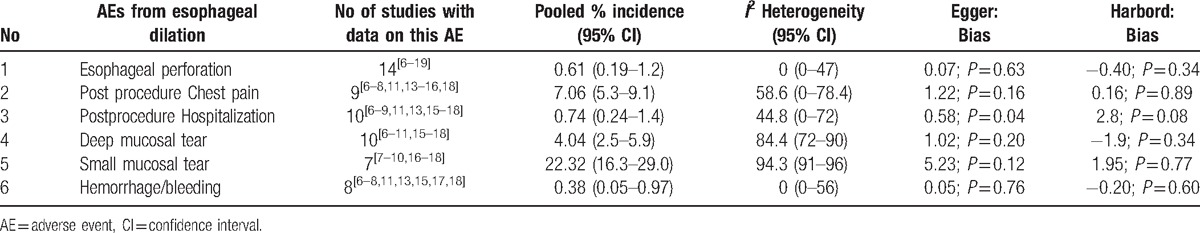
Pooled percentage of individual complications post procedure, bias, and heterogeneity.

## 4 Discussion

EoE is a chronic inflammatory condition characterized with marked infiltration of esophageal mucosa with eosinophils. The common symptoms include dysphagia and recurrent food impactions. There are multiple treatment modalities including avoidance of allergen foods, swallowed glucocorticoids, and frequent esophageal dilations. As mentioned previously, endoscopic esophageal dilation has specific role in EoE patients with esophageal strictures that result in dysphagia. Individual studies have been published assessing the safety and efficacy of endoscopic dilation in EoE patients. The results of most of these studies were not uniform.

In a retrospective study by Ally et al, 196 patients with EoE were identified, out of which 54 patients (28%) had a total of 66 dilations and there were no major complications involved. Two patients (4%) had chest pain, which was a minimal complication.^[7]^ In a cohort study by Schoepfer et al, total of 207 patients with EoE treated with esophageal dilation were included. Esophageal dilation alone or in combination with antieosinophilic medication was highly effective in providing long-lasting symptom relief up to >20 months; however, it did not influence the underlying inflammatory process. There were no major complications, but 74% patients reported postdilation chest pain.^[13]^ In another cross-sectional analysis by Bohm et al, 16 adult EoE patients were included and esophageal dilation was performed in 13 patients (81%). Ten patients (77%) had long-lasting symptomatic relief for an average of 2 years. There was no impact on the mucosal eosinophilia or gross endoscopy findings.^[14]^

In a retrospective study by Enns et al^[12]^, 80% of patients had symptom relief and no complications with esophageal dilation. However, 83% had symptom recurrence in 1 year. In a relatively large retrospective study by Dellon et al^[11]^, 36 of 130 patients had dilations with 83% response rate. Seven percent of patients had minor complications like chest pain or mucosal tears, but no major complications were reported. All complications occurred in patients with simultaneous topical steroids.

Multiple retrospective studies were done in the recent years, which demonstrated that esophageal dilation is a safe treatment option for patients with EoE. Multiple dilations may be required for symptom recurrence. Esophageal dilation is commonly associated with minor complications like chest pain and mucosal tears. However, severe complications like perforation and uncontrollable hemorrhage are very rare. All the perforations found in these studies were managed medically without needing surgical intervention. Patients with baseline narrowed esophagus required multiple dilations for symptom relief. Patients with proximal strictures and strictures preventing initial endoscope passage were found to be at increased risk for severe complications. Serial dilations and careful selection of the initial dilator size guided by repeated endoscopic inspections to look for tears were deemed to be safe practice methods.^[6,15–19]^ When serial dilations are required, it is a common practice to not increase the dilation by >3 mm in the subsequent procedures.^[15]^ This would necessitate multiple dilations to attain a target esophageal diameter of 15 to 18 mm.^[13,14,31]^

This meta-analysis suggests that esophageal dilation is associated with clinical improvement in 84.95% of pooled patients, after the median follow-up period of 12 months. This is much higher than the clinical improvement that was analyzed in a previous meta-analysis in 2013, by Moawad et al.^[4]^ In patients with confirmed diagnosis of EoE, endoscopic esophageal dilation seems to be a safe management option. The common complications were chest pain and small mucosal tears, which occurred in 7.06% and 22.32% of pooled patients, respectively. The severe and rare complications were esophageal perforations, hemorrhage, and deep mucosal tears, which accounted for about 0.81%, 0.38%, and 4.04% of the pooled population, respectively. The hospitalization rates for the pooled population were 0.74%. There were no procedure-related deaths. Majority patients with chest pain and deep mucosal tears did not require hospitalization and symptoms were self-limiting.

This is an up-to-date meta-analysis that includes all the studies in the field of EoE assessing the efficacy and complications of endoscopic esophageal dilation. Based on the aforementioned results and the previous studies, esophageal dilation seems to be a safe and effective treatment modality for EoE irrespective of the usage of the topical steroids.

Strengths of this meta-analysis include the high-quality methodology of statistical analysis, high-quality methodology used in individual studies, large number of studies that met the inclusion criteria, and total number of patients included in this analysis (N = 1607).

### 4.1 Limitations

Unfortunately, there are no prospective studies or randomized controlled trials evaluating the role of endoscopic esophageal dilation in EoE patients. All the studies that met the inclusion criteria were retrospective studies. This inherently introduces bias into this analysis. We were unable to perform cost–benefit analysis because of the lack of data from individual studies. Local expertise plays a key role in the outcomes of endoscopic dilation procedures. As the studies were performed in different countries, using different endoscope equipment, used by different operators with varying skill sets, this variable should be considered while we try to analyze the final outcomes. Owing to the nature of data available from individual studies, we were not able to analyze and compare various types of esophageal dilation (savary, TTS, and Maloney). The individual studies included in this meta-analysis were performed in highly specialized and famous worldwide centers, and this maybe a reason for higher success and lower complication rates of EoE dilation procedure.

In treatment of EoE patients, granted there is operator expertise and infrastructural availability, endoscopic esophageal dilation is a safe and excellent management option, and should be used in the following clinical scenarios:

- EoE patients with dysphagia secondary to esophageal strictures and rings.
- For EoE patient who failed conservative therapy and as initial therapy for patient with high grade strictures and dysphagia on presentation.

Operator expertise and availability of specific tools should be a guide in selecting one technique over the other. Currently, esophageal dilation is restricted to specialized centers and not widely available. There is a need for randomized controlled trials to further solidify the currently available evidence. Further, studies should be performed looking at quality of life and cost–benefit analysis.

Studies with statistically significant positive results tend to be published and cited. Additionally, smaller studies may show larger treatment effects compared to larger studies. This publication and selection bias may affect the summary estimates. The bias can be estimated using Egger bias indicators and the construction of funnel plots, whose shape can be affected by bias. In the present meta-analysis and systematic review, bias calculations both Egger ^[27]^ and Begg-Mazumdar,^[28]^ bias indicators showed no statistically significant bias. Furthermore, analysis using funnel plots showed no significant publication bias among the studies included in the present analysis.

## 5 Conclusion

In patients with a confirmed diagnosis of EoE, endoscopic esophageal dilation seems to be a safe and effective management option for patients with dysphagia from esophageal strictures/rings who failed conservative management.
